# Physical contact in physical education for women: A cross-sectional questionnaire-based study of discomfort related to initiator gender, relationship with the initiator, and body region

**DOI:** 10.1371/journal.pone.0358338

**Published:** 2026-09-11

**Authors:** Megumi Gonno, Noriyuki Kida

**Affiliations:** 1 Department of Childhood Education, Faculty of Childhood Education, Nagoya Aoi University, Nagoya, Japan; 2 Faculty of Arts and Sciences, Kyoto Institute of Technology, Hashikami-cho, Matsugasaki, Sakyo-ku, Kyoto, Japan; University of Bamberg, GERMANY

## Abstract

Physical contact is frequently used in sports instruction, but perceptions of discomfort may vary according to social and contextual factors. In this study, discomfort toward physical contact was examined using two parallel designs: one focusing on teachers as initiators in relation to initiator gender and body region, and the other focusing on initiators without a specified function in relation to initiator gender, relationship (close friend or acquaintance), and body region. A cross-sectional questionnaire study was conducted among 175 female university students in Japan. Participants evaluated 66 hypothetical scenarios comprising 22 teacher-initiated conditions (2 initiator genders × 11 body regions) and 44 conditions involving close friends or acquaintances (2 initiator genders × 2 relationship levels × 11 body regions). Discomfort was assessed as a binary outcome (yes/no). Data were analyzed using separate binary logistic regression models for the two designs: initiator gender × body region for teacher-initiated contact, and initiator gender × relationship × body region for contact initiated by close friends or acquaintances. For teacher-initiated contact, significant main effects of initiator gender and body region were observed, whereas the initiator gender × body region interaction was not significant. For contact initiated by close friends or acquaintances, significant main effects of initiator gender, relationship, and body region were observed, together with a significant initiator gender × relationship interaction; interactions involving body region were not significant. Across both designs, discomfort was greater for male initiators and varied across body regions. Among close friends and acquaintances, discomfort was greater for male initiators in both relationship conditions, but the estimated gender contrast was larger for close friends than for acquaintances. These findings suggest that physical contact in sports-related contexts should be considered in relation to initiator gender and body region, as well as relational closeness when the initiator’s function is not specified.

## 1. Introduction

Physical contact is frequently used as an instructional tool in sports coaching and physical education, serving functions such as technique demonstration, movement correction, and physical support. However, it also raises concerns related to psychological discomfort, boundary management, and safeguarding, particularly in contexts involving asymmetrical relationships such as those between coaches and athletes. Previous research has consistently shown that responses to interpersonal touch are shaped by social and contextual factors rather than by functional intent alone. In particular, three factors have been identified as key determinants of how touch is perceived: the gender of the person initiating the contact, the relational closeness between the individuals involved, and the body region in question [1–4]. For example, opposite-gender contact and contact from less familiar individuals are generally perceived as more uncomfortable, especially for body regions associated with higher levels of intimacy. These findings suggest that the acceptability of touch is systematically structured by social and physical factors.

However, most of this evidence has been obtained from general interpersonal contexts rather than educational or coaching settings. In the field of sports instruction, physical contact between coaches and athletes is often used for purposes such as technical guidance, support, and encouragement. Concerns regarding psychological resistance and discomfort associated with such contact have received increasing attention in recent research [5,6], but there is relatively little research regarding the perception of touch in sports-specific instructional contexts. As a result, it remains unclear how factors such as initiator gender, relational closeness, and body region operate in sports-specific contexts, particularly when physical contact occurs within instructional settings where it may be functionally justified and socially legitimized. In such settings, additional elements, including authority relationships and instructional norms, may further influence how touch is perceived. Although initiator gender, relational closeness, and body region have been examined individually, relatively little is known about how these factors shape discomfort in sports-specific contexts, particularly when physical contact occurs as part of instruction. It also remains unclear whether the effects of initiator gender and body region are consistent across different social contexts. This gap limits our understanding of how social and bodily factors shape discomfort toward physical contact in situations where such contact is pedagogically embedded.

The present study addresses the gap just outlined by examining discomfort toward physical contact in sports-related contexts in relation to initiator gender, relational closeness, and body region, while also considering the specific case of teacher-initiated contact. Using a questionnaire-based within-subject design, we examine the effects of initiator gender and body region for teacher-initiated contact and the effects of initiator gender, relational closeness, and body region for contact initiated by close friends or acquaintances.

## 2. Materials and methods

### 2.1. Study design and study setting

A cross-sectional questionnaire study without any between-subject factors was conducted in five undergraduate courses related to sports and physical activity within a teacher-training curriculum at a university in Japan. These courses were offered within departments of childhood and early childhood education and were designed to prepare students for careers in education and childcare. The courses included a first-year sports science course, two second-year courses focusing on physical activity and childcare practice, a third-year course involving model teaching sessions for elementary school physical education, and a seminar-style course. All courses were taught by one of the authors (M.G.), and data collection was conducted during regular class sessions.

### 2.2. Questionnaire

Data were collected using a questionnaire developed for the present study to assess discomfort toward physical contact in sports-related contexts. The questionnaire comprised 66 hypothetical scenarios. For analysis and interpretation, these scenarios were organized into two parallel designs. The first design comprised 22 teacher-initiated scenarios varying initiator gender (male or female) and body region (11 areas). The second design comprised 44 scenarios in which the initiator’s function was not specified, varying initiator gender (male or female), relational closeness, and body region (11 areas). In the original questionnaire, the two relational-closeness conditions were described as a “friend (someone close to you)” and “someone you are not very close to”; for conciseness, these conditions are referred to hereafter as “close friend” and “acquaintance,” respectively. This distinction was made because “teacher” specifies the function of the initiator, whereas “close friend” and “acquaintance” specify relational closeness; accordingly, teacher was not treated as a level of the relationship factor in the analyses. Participants were instructed to imagine being touched during a sports activity and to indicate whether they would feel discomfort in each scenario. Discomfort was assessed as a binary response (yes/no). The questionnaire was developed based on previous research [7] and adapted for sports instructional contexts. Feedback from researchers in psychology and sports science was used to refine item wording and improve content validity. The English translation of the questionnaire and the original Japanese questionnaire are provided as Supporting Information (S1 File and S2 File, respectively).

### 2.3. Participant recruitment and study procedures

Participants were recruited from the target classes during regular class sessions conducted between April 8 and April 16, 2025. Eligibility criteria included enrollment in one of the target courses and age 18 years or older. Participation was voluntary and unpaid. After receiving an explanation of the study, participants provided written informed consent and completed the questionnaire in paper-and-pencil format during class. The questionnaire was administered by one of the authors (M.G.), who also served as the course instructor. Participants’ names were collected solely to enable withdrawal of consent during the designated withdrawal period and were not used in the analysis. After the withdrawal period had ended, identifying information was removed so that individual participants could no longer be identified from the research dataset. All participants completed all questionnaire items; therefore, no missing data were observed.

### 2.4. Statistical analysis

Data were analyzed using ordinary binary logistic regression because discomfort was coded as a binary outcome (yes/no). In accordance with the two-design structure described above, separate analyses were conducted for teacher-initiated contact and for contact initiated by close friends or acquaintances. All predictors included in each model were treated as categorical variables.

For teacher-initiated contact, the full logistic regression model included the main effects of initiator gender and body region and their two-way interaction (initiator gender × body region). For contact initiated by close friends or acquaintances, the full logistic regression model included the main effects of initiator gender, relationship (close friend vs. acquaintance), and body region, all two-way interactions (initiator gender × relationship, initiator gender × body region, and relationship × body region), and the three-way interaction (initiator gender × relationship × body region). Statistical significance of each effect was evaluated using Wald chi-square tests derived from the corresponding full model.

To obtain readily interpretable regression coefficients, odds ratios (ORs), and 95% confidence intervals (CIs) for each predictor, separate binary logistic regression models were additionally fitted with only the corresponding predictor included in the model. Because a significant initiator gender × relationship interaction was observed in the close friend/acquaintance design, follow-up binary logistic regression analyses were conducted separately within the close-friend and acquaintance conditions to estimate the effect of initiator gender.

Statistical significance was set at p < 0.05. All analyses were conducted using IBM SPSS Statistics Version 28 (IBM Corp., Armonk, NY, USA).

### 2.5. Ethical considerations

This study was approved by the Nagoya Women’s University Ethics Committee for Human Research (Approval No. 2024−29; approval date: March 28, 2024). All participants provided written informed consent prior to participation.

## 3. Results

### 3.1. Participant characteristics

A total of 179 students participated in the study. Four male participants were excluded because the present study focuses on female students’ perceptions of physical contact. The final analytic sample consisted of 175 female university students (mean age = 18.7 years, SD = 0.8, range = 18–22 years). The sample included 82 first-year, 66 second-year, and 27 third-year students.

### 3.2. Results for teachers as initiators

In the teacher-initiated contact design, significant main effects of initiator gender and body region were observed, whereas the initiator gender × body region interaction was not significant (Table 1). Discomfort was higher for male than for female teachers and varied across body regions, with the highest marginal percentages observed for the chest and buttocks and the lowest for the arm and hand (Table 2).

**Table 1:** Effects of initiator gender and body region on discomfort in teacher-initiated physical contact (N = 175).

Effect	Wald χ²	df	p
Initiator gender	641.580	1	< 0.001
Body region	359.611	10	< 0.001
Initiator gender × Body region	5.806	10	0.831

Note. Wald chi-square statistics were obtained from a full binary logistic regression model including the main effects of initiator gender and body region and their two-way interaction. df indicates the degrees of freedom for each Wald chi-square test.

**Table 2:** Percentages of discomfort and logistic regression coefficients for initiator gender and body region in teacher-initiated physical contact (N = 175).

Factor	Category	Discomfort %	B	OR	95% CI	p
Initiator gender	Female (Ref.)	21.8				
	Male	64.2	1.861	6.433	5.577–7.421	< 0.001
Body region	Hand (Ref.)	23.7				
	Arm	22.9	−0.048	0.953	0.671–1.353	0.788
	Head	32.0	0.415	1.514	1.085–2.113	0.015
	Back	32.9	0.454	1.574	1.129–2.195	0.007
	Neck/Shoulders	34.3	0.518	1.678	1.206–2.336	0.002
	Leg/Foot	39.1	0.727	2.069	1.492–2.869	< 0.001
	Lower Back	46.0	1.008	2.740	1.982–3.788	< 0.001
	Face	50.0	1.168	3.217	2.328–4.445	< 0.001
	Abdomen	53.4	1.306	3.691	2.670–5.101	< 0.001
	Buttocks	67.4	1.896	6.659	4.775–9.288	< 0.001
	Chest	70.9	2.057	7.821	5.582–10.960	< 0.001

Note. Percentages represent the marginal proportion of responses indicating discomfort within each category, collapsing across the other factor in the teacher condition. Logistic regression coefficients (B), odds ratios (ORs), 95% confidence intervals (CIs), and p-values were obtained from separate binary logistic regression models containing only the corresponding factor as a predictor. Female initiator and hand served as the reference categories.

### 3.3. Results for initiators without a specified function

In the close friend/acquaintance design, significant main effects of initiator gender, relationship, and body region were observed. The initiator gender × relationship interaction was also significant, whereas the initiator gender × body region interaction, the relationship × body region interaction, and the three-way interaction were not significant (Table 3). Overall, the marginal percentage of discomfort was higher for male than for female initiators and for acquaintances than for close friends, and varied across body regions (Table 4). Follow-up analyses of the significant initiator gender × relationship interaction indicated that discomfort was higher for male initiators in both relationship conditions, but the estimated gender contrast was larger for close friends than for acquaintances (Table 5).

**Table 3:** Effects of initiator gender, relationship, and body region on discomfort in physical contact initiated by close friends and acquaintances (N = 175).

Effect	Wald χ²	df	p
Initiator gender	918.188	1	< 0.001
Relationship	400.136	1	< 0.001
Body region	623.326	10	< 0.001
Initiator gender × Relationship	26.950	1	< 0.001
Initiator gender × Body region	11.830	10	0.297
Relationship × Body region	12.194	10	0.272
Initiator gender × Relationship × Body region	6.501	10	0.772

Note. Wald chi-square statistics were obtained from a full binary logistic regression model including the main effects of initiator gender, relationship, and body region, all two-way interactions, and the three-way interaction. The relationship factor comprised close friend and acquaintance. df indicates the degrees of freedom for each Wald chi-square test.

**Table 4:** Percentages of discomfort and logistic regression coefficients for initiator gender, relationship, and body region in physical contact initiated by close friends and acquaintances (N = 175).

Factor	Category	Discomfort %	B	OR	95% CI	p
Initiator gender	Female (Ref.)	22.9				
	Male	59.5	1.600	4.954	4.487–5.470	< 0.001
Relationship	Close friend (Ref.)	30.3				
	Acquaintance	52.1	0.918	2.505	2.281–2.750	< 0.001
Body region	Hand (Ref.)	21.6				
	Arm	23.0	0.083	1.086	0.844–1.397	0.521
	Back	29.6	0.423	1.527	1.198–1.945	< 0.001
	Head	30.4	0.464	1.590	1.249–2.025	< 0.001
	Neck/Shoulders	33.6	0.608	1.837	1.447–2.333	< 0.001
	Leg/Foot	40.0	0.885	2.424	1.916–3.066	< 0.001
	Lower Back	43.3	1.021	2.775	2.196–3.507	< 0.001
	Face	45.9	1.125	3.079	2.438–3.889	< 0.001
	Abdomen	52.3	1.382	3.984	3.155–5.031	< 0.001
	Buttocks	63.6	1.848	6.345	5.006–8.041	< 0.001
	Chest	69.7	2.125	8.369	6.572–10.658	< 0.001

Note. Percentages represent the marginal proportion of responses indicating discomfort within each category, collapsing across the other factors in the close friend/acquaintance design. Logistic regression coefficients (B), odds ratios (ORs), 95% confidence intervals (CIs), and p-values were obtained from separate binary logistic regression models containing only the corresponding factor as a predictor. Female initiator, close friend, and hand served as the reference categories.

**Table 5:** Follow-up analyses of initiator gender within each relationship condition in physical contact initiated by close friends and acquaintances (N = 175).

Relationship	Discomfort %		Contrast male vs female			
	Female	Male	B	OR	95% CI	p
Close friend	11.3	49.2	2.026	7.582	6.417–8.959	< 0.001
Acquaintance	34.4	69.8	1.482	4.402	3.845–5.040	< 0.001

Note. Percentages represent discomfort within each initiator gender × relationship condition. Because the initiator gender × relationship interaction was significant in the full model (Table 3), follow-up analyses estimated the effect of initiator gender separately for close friends and acquaintances, with female initiator as the reference category.

## 4. Discussion

### 4.1. Summary of findings

In the present study, discomfort toward physical contact in sports-related contexts was examined using two parallel designs that distinguished teacher-initiated contact from contact initiated by close friends or acquaintances. In the teacher-initiated contact design, discomfort differed significantly according to initiator gender and body region, whereas the initiator gender × body region interaction was not significant. In the close friend/acquaintance design, significant main effects of initiator gender, relational closeness, and body region were observed, together with a significant initiator gender × relationship interaction. Follow-up analyses indicated that discomfort was higher for male initiators in both relationship conditions, but the estimated gender contrast was larger for close friends than for acquaintances. Interactions involving body region were not significant in either design. Taken together, these findings indicate that initiator gender and body region are relevant to discomfort across both designs, while relational closeness is additionally associated with discomfort when the initiator’s function is not specified.

### 4.2. Initiator gender and body region across the two designs

Across both designs, discomfort was greater for male than for female initiators and varied across body regions. In both the teacher-initiated and close friend/acquaintance designs, the chest and buttocks were among the regions associated with the highest levels of discomfort, whereas the hand and arm were among those associated with the lowest levels. These findings are consistent with previous studies reporting gender differences in responses to interpersonal touch [2,3,8] and systematic variation in the acceptability of touch across body regions [4,9,10]. The present findings suggest that these factors are also relevant in sports-related contexts, including situations in which physical contact is initiated by a teacher. The initiator gender × body region interaction was not significant in either design. This suggests that although overall discomfort differed between male and female initiators, the relative pattern of discomfort across body regions was broadly similar for both initiator genders. Thus, the association between body region and discomfort appears to be relatively stable across initiator gender in both teacher-initiated contact and contact initiated by close friends or acquaintances.

### 4.3. Relational closeness and the initiator gender × relationship interaction

In the close friend/acquaintance design, discomfort was always higher for acquaintances than for close friends. This finding is consistent with previous research showing that perceptions and acceptance of interpersonal touch vary with relational closeness and emotional bonds [4,5,11,12]. Thus, even within sports-related contexts, the social relationship between the individuals involved appears to be relevant to how physical contact is perceived.

However, the significant initiator gender × relationship interaction indicates that the association between initiator gender and discomfort differed in magnitude according to relational closeness. The follow-up analyses showed that discomfort was higher for male than for female initiators in both the close-friend and acquaintance conditions, but the estimated gender contrast was larger for close friends than for acquaintances. This interaction only represents a difference in the magnitude of the gender contrast but no reversal of its direction, i.e., in both relationship conditions, male-initiated contact was associated with greater discomfort. One possible interpretation is that close relational ties may be associated with particularly low discomfort for female-initiated contact, whereas the reduction in discomfort associated with closeness may be less pronounced for male-initiated contact. As variables such as trust, perceived intimacy, or the meaning attributed to the contact were not assessed in the present study, this interpretation should be considered tentative.

### 4.4. Practical implications for sports instruction and physical education

The present findings have practical implications for the use of physical contact in sports instruction and physical education. In the teacher-initiated contact design, discomfort was higher for male than for female teachers and was particularly high for contact involving the chest and buttocks. These findings suggest that instructors should not assume that physical contact will be perceived as acceptable simply because it serves an instructional purpose. When physical contact is considered necessary for technical guidance, movement correction, or physical support, instructors should take into account both the body region involved and the possibility that perceptions may differ according to initiator gender. From a practical perspective, whenever feasible, explaining the purpose of the contact beforehand and confirming the participant’s willingness to receive such contact may help in avoiding unwanted or unexpected physical contact.

The findings for close friends and acquaintances further indicate that relational closeness is relevant but should not be assumed to eliminate discomfort. Although discomfort was lower overall for close friends than for acquaintances, male-initiated contact was associated with greater discomfort in both relationship conditions. Moreover, because interactions involving body region were not significant, the relative pattern of more and less sensitive body regions appeared broadly similar across initiator gender and relational closeness. This suggests that practical guidance concerning contact with more sensitive body regions may be applicable across a range of interpersonal contexts rather than requiring entirely different rules for each combination of initiator gender and relationship. Nevertheless, individual preferences are likely to vary, and explicit communication and respect for personal boundaries remain important when physical contact is used in sports-related settings.

These practical considerations are consistent with broader efforts to manage physical contact in structured instructional settings. Formal policies addressing appropriate physical contact have been developed in some performing arts settings, including ballet [13,14]. In sports, research on safeguarding in coach–athlete relationships and on the relational role of touch in coaching has likewise highlighted the importance of attending to the interpersonal context in which physical contact occurs [15,16]. Together, these perspectives support the development of context-sensitive practices that balance instructional needs with participants’ comfort and personal boundaries.

### 4.5. Limitations and future directions

Several limitations should be considered when interpreting the present findings. First, the participants were female university students enrolled in teacher-training courses at a single university in Japan. The findings therefore cannot necessarily be generalized to female athletes, students in other educational settings, individuals of different ages or genders, or populations in other cultural contexts. To examine this, similar studies should be conducted with more diverse samples, including athletes and participants in actual sports and physical education settings.

Second, the study assessed responses to hypothetical scenarios rather than experiences of actual physical contact. Although this approach allowed the same combinations of initiator characteristics and body regions to be evaluated systematically, anticipated discomfort may not fully correspond to discomfort experienced during actual interactions. In real-world settings, perceptions of physical contact may also depend on factors not assessed in the present study, including the purpose and necessity of the contact, prior explanation, situational context, trust, and previous experiences with the initiator. Future research should incorporate these contextual factors and examine physical-contact experiences in more ecologically valid sports and physical education settings.

Third, the questionnaire distinguished teacher-initiated contact from contact initiated by close friends or acquaintances because teacher represents the initiator’s function, whereas close friend and acquaintance represent relational closeness. However, the present questionnaire did not independently manipulate initiator function and relational closeness. Consequently, the study cannot determine how relational closeness with a teacher, or the specific function of a close friend or acquaintance, may influence discomfort. Future studies employing designs that independently vary initiator function and relational closeness would help clarify their respective and combined associations with perceptions of physical contact.

Finally, discomfort was assessed using a binary response format. This facilitated straightforward interpretation but did not capture differences in the degree or intensity of discomfort. Future studies could use graded response scales and examine additional psychological responses, such as perceived appropriateness, acceptability, safety, or trust. Such work may provide a more detailed understanding of the conditions under which physical contact is perceived as acceptable or uncomfortable in sports-related settings.

## 5. Conclusions

In the present study, discomfort toward physical contact in sports-related contexts was examined using two parallel designs that distinguished teacher-initiated contact from contact initiated by close friends or acquaintances. In teacher-initiated contact, discomfort was associated with initiator gender and body region, whereas in contact initiated by close friends or acquaintances, initiator gender, relational closeness, and body region were associated with discomfort. Male-initiated contact was associated with greater discomfort across both designs, and discomfort varied across body regions in both designs. Among close friends and acquaintances, the gender contrast was larger for close friends than for acquaintances, although discomfort was greater for male initiators in both relationship conditions. These findings underscore the importance of considering initiator gender and body region when physical contact is used in sports-related settings, as well as relational closeness when the initiator’s function is not specified. In sports instruction and physical education, careful communication and respect for individual boundaries are important when physical contact is used.

## Supporting information

S1 File English translation of the questionnaire. (DOCX)

S2 File Original Japanese questionnaire. (DOCX)
