# Supplementary material for: Physical contact in physical education for women: A cross-sectional questionnaire-based study of discomfort related to initiator gender, relationship with the initiator, and body region
Source: PLoS One. 2026 Sep 11;21(9):e0358338. doi: 10.1371/journal.pone.0358338 (PMC13567764; doi:10.1371/journal.pone.0358338)
Supplement: Supplementary file 1 [file pone.0358338.s001.docx]

**S1 File. English translation of the questionnaire**

*Supporting Information for*

**Physical Contact in Physical Education for Women: A Cross-Sectional Questionnaire-Based Study of Discomfort Related to Initiator Gender, Relationship with the Initiator, and Body Region**

**Supporting Information note (not part of the administered questionnaire).** This file is an English translation of the original Japanese questionnaire. The name field was included solely to allow participants to withdraw consent during the designated withdrawal period. Names were not used in the analysis, and identifying information was removed after the withdrawal period. In the administered questionnaire, initiator sex was presented as “same sex” and “opposite sex.” Because the analytic sample comprised only women, these categories corresponded to female and male initiators, respectively, in the analyses. The English wording below follows the original Japanese questionnaire; formatting has been standardized for readability.

**Questionnaire (English translation)**

Date administered: ____________________	Name: ______________________________

Age: (	) years

Sex: Male	/	Female	/	Prefer not to answer

Experience in sports-related clubs and/or lessons (including dance): Yes	/	No

Frequency of physical contact with others in daily life: Very often	/	Somewhat often	/	Neither	/	Not very often	/	Almost never

In the following questions, the body is divided into 11 regions (1–11). For each type of relationship, please imagine how you would feel if that person touched each body region during a sports class and indicate your response.

**Friend (someone you are close to)**

| **Same sex** | **Opposite sex** |
| --- | --- |
| 1. Head ( Uncomfortable / Not uncomfortable )  2. Face ( Uncomfortable / Not uncomfortable )  3. Neck + shoulders ( Uncomfortable / Not uncomfortable )  4. Chest ( Uncomfortable / Not uncomfortable )  5. Abdomen ( Uncomfortable / Not uncomfortable )  6. Back ( Uncomfortable / Not uncomfortable )  7. Lower back ( Uncomfortable / Not uncomfortable )  8. Buttocks ( Uncomfortable / Not uncomfortable )  9. Arm (upper arm + forearm) ( Uncomfortable / Not uncomfortable )  10. Hand ( Uncomfortable / Not uncomfortable )  11. Leg + foot ( Uncomfortable / Not uncomfortable ) | 1. Head ( Uncomfortable / Not uncomfortable )  2. Face ( Uncomfortable / Not uncomfortable )  3. Neck + shoulders ( Uncomfortable / Not uncomfortable )  4. Chest ( Uncomfortable / Not uncomfortable )  5. Abdomen ( Uncomfortable / Not uncomfortable )  6. Back ( Uncomfortable / Not uncomfortable )  7. Lower back ( Uncomfortable / Not uncomfortable )  8. Buttocks ( Uncomfortable / Not uncomfortable )  9. Arm (upper arm + forearm) ( Uncomfortable / Not uncomfortable )  10. Hand ( Uncomfortable / Not uncomfortable )  11. Leg + foot ( Uncomfortable / Not uncomfortable ) |

**Someone you are not very close to**

| **Same sex** | **Opposite sex** |
| --- | --- |
| 1. Head ( Uncomfortable / Not uncomfortable )  2. Face ( Uncomfortable / Not uncomfortable )  3. Neck + shoulders ( Uncomfortable / Not uncomfortable )  4. Chest ( Uncomfortable / Not uncomfortable )  5. Abdomen ( Uncomfortable / Not uncomfortable )  6. Back ( Uncomfortable / Not uncomfortable )  7. Lower back ( Uncomfortable / Not uncomfortable )  8. Buttocks ( Uncomfortable / Not uncomfortable )  9. Arm (upper arm + forearm) ( Uncomfortable / Not uncomfortable )  10. Hand ( Uncomfortable / Not uncomfortable )  11. Leg + foot ( Uncomfortable / Not uncomfortable ) | 1. Head ( Uncomfortable / Not uncomfortable )  2. Face ( Uncomfortable / Not uncomfortable )  3. Neck + shoulders ( Uncomfortable / Not uncomfortable )  4. Chest ( Uncomfortable / Not uncomfortable )  5. Abdomen ( Uncomfortable / Not uncomfortable )  6. Back ( Uncomfortable / Not uncomfortable )  7. Lower back ( Uncomfortable / Not uncomfortable )  8. Buttocks ( Uncomfortable / Not uncomfortable )  9. Arm (upper arm + forearm) ( Uncomfortable / Not uncomfortable )  10. Hand ( Uncomfortable / Not uncomfortable )  11. Leg + foot ( Uncomfortable / Not uncomfortable ) |

**Teacher**

| **Same sex** | **Opposite sex** |
| --- | --- |
| 1. Head ( Uncomfortable / Not uncomfortable )  2. Face ( Uncomfortable / Not uncomfortable )  3. Neck + shoulders ( Uncomfortable / Not uncomfortable )  4. Chest ( Uncomfortable / Not uncomfortable )  5. Abdomen ( Uncomfortable / Not uncomfortable )  6. Back ( Uncomfortable / Not uncomfortable )  7. Lower back ( Uncomfortable / Not uncomfortable )  8. Buttocks ( Uncomfortable / Not uncomfortable )  9. Arm (upper arm + forearm) ( Uncomfortable / Not uncomfortable )  10. Hand ( Uncomfortable / Not uncomfortable )  11. Leg + foot ( Uncomfortable / Not uncomfortable ) | 1. Head ( Uncomfortable / Not uncomfortable )  2. Face ( Uncomfortable / Not uncomfortable )  3. Neck + shoulders ( Uncomfortable / Not uncomfortable )  4. Chest ( Uncomfortable / Not uncomfortable )  5. Abdomen ( Uncomfortable / Not uncomfortable )  6. Back ( Uncomfortable / Not uncomfortable )  7. Lower back ( Uncomfortable / Not uncomfortable )  8. Buttocks ( Uncomfortable / Not uncomfortable )  9. Arm (upper arm + forearm) ( Uncomfortable / Not uncomfortable )  10. Hand ( Uncomfortable / Not uncomfortable )  11. Leg + foot ( Uncomfortable / Not uncomfortable ) |

*This is the end of the questionnaire. Thank you for your cooperation.*
