# Supplementary material for: Physical contact in physical education for women: A cross-sectional questionnaire-based study of discomfort related to initiator gender, relationship with the initiator, and body region
Source: PLoS One. 2026 Sep 11;21(9):e0358338. doi: 10.1371/journal.pone.0358338 (PMC13567764; doi:10.1371/journal.pone.0358338)
Supplement: Supplementary file 2 [file pone.0358338.s002.docx]

**S2 File. Original Japanese questionnaire**

*Supporting Information for*

**Physical Contact in Physical Education for Women: A Cross-Sectional Questionnaire-Based Study of Discomfort Related to Initiator Gender, Relationship with the Initiator, and Body Region**

**Supporting Information 注記（調査時の質問紙には含まれない）**
本ファイルには、調査時に使用した日本語質問紙を収録している。氏名欄は、所定の同意撤回期間内に研究参加への同意を撤回できるようにする目的で設けたものであり、氏名は分析には使用していない。同意撤回期間終了後、研究データから個人を特定できる情報を除外した。本研究の解析対象は女性のみであったため、質問紙の「同性」「異性」は、分析上それぞれ女性の接触者、男性の接触者に対応した。

実施日：　　　　　　　　　　　　　　　　氏名：

・年齢　（　　　）歳

・性別　（　男性　・　女性　・　回答しない　）

・運動系の部活動および習い事経験の有無（ダンスなども含む）（　ある　・　ない　）

・日常生活での他者との身体接触頻度

（非常にある　・　ややある　・　どちらでもない　・　あまりない　・　ほとんどない）

・以下の質問では、身体の部位を①～⑪に分けています。それぞれの関係性の相手に各部位をスポーツ授業時に触られた場合の感情を想定し回答してください。

**友人（親しい人）**

**【同性】**

　① **あたま**　（　不快　・　不快でない　）

　② **かお**　　（　不快　・　不快でない　）

　③ **くび＋かた**（　不快　・　不快でない　）

　④ **むね**　　（　不快　・　不快でない　）

　⑤ **おなか**　（　不快　・　不快でない　）

　⑥ **せなか**　（　不快　・　不快でない　）

　⑦ **こし**　　（　不快　・　不快でない　）

　⑧ **おしり**　（　不快　・　不快でない　）

　⑨ **うで（上腕＋前腕）**　（　不快　・　不快でない　）

　⑩ **て**　　（　不快　・　不快でない　）

　⑪ **あし（脚＋足）**　（　不快　・　不快でない　）

**【異性】**

① **あたま**　（　不快　・　不快でない　）

　② **かお**　　（　不快　・　不快でない　）

　③ **くび＋かた**（　不快　・　不快でない　）

　④ **むね**　　（　不快　・　不快でない　）

　⑤ **おなか**　（　不快　・　不快でない　）

　⑥ **せなか**　（　不快　・　不快でない　）

　⑦ **こし**　　（　不快　・　不快でない　）

　⑧ **おしり**　（　不快　・　不快でない　）

　⑨ **うで（上腕＋前腕）**　（　不快　・　不快でない　）

　⑩ **て**　　（　不快　・　不快でない　）

　⑪ **あし（脚＋足）**　（　不快　・　不快でない　）

**あまり親しくない人**

**【同性】**

① **あたま**　（　不快　・　不快でない　）

　② **かお**　　（　不快　・　不快でない　）

　③ **くび＋かた**（　不快　・　不快でない　）

　④ **むね**　　（　不快　・　不快でない　）

　⑤ **おなか**　（　不快　・　不快でない　）

　⑥ **せなか**　（　不快　・　不快でない　）

　⑦ **こし**　　（　不快　・　不快でない　）

　⑧ **おしり**　（　不快　・　不快でない　）

　⑨ **うで（上腕＋前腕）**　（　不快　・　不快でない　）

　⑩ **て**　　（　不快　・　不快でない　）

　⑪ **あし（脚＋足）**　（　不快　・　不快でない　）

**【異性】**

① **あたま**　（　不快　・　不快でない　）

　② **かお**　　（　不快　・　不快でない　）

　③ **くび＋かた**（　不快　・　不快でない　）

　④ **むね**　　（　不快　・　不快でない　）

　⑤ **おなか**　（　不快　・　不快でない　）

　⑥ **せなか**　（　不快　・　不快でない　）

　⑦ **こし**　　（　不快　・　不快でない　）

　⑧ **おしり**　（　不快　・　不快でない　）

　⑨ **うで（上腕＋前腕）**　（　不快　・　不快でない　）

　⑩ **て**　　（　不快　・　不快でない　）

　⑪ **あし（脚＋足）**　（　不快　・　不快でない　）

**教員**

**【同性】**

① **あたま**　（　不快　・　不快でない　）

　② **かお**　　（　不快　・　不快でない　）

　③ **くび＋かた**（　不快　・　不快でない　）

　④ **むね**　　（　不快　・　不快でない　）

　⑤ **おなか**　（　不快　・　不快でない　）

　⑥ **せなか**　（　不快　・　不快でない　）

　⑦ **こし**　　（　不快　・　不快でない　）

　⑧ **おしり**　（　不快　・　不快でない　）

　⑨ **うで（上腕＋前腕）**　（　不快　・　不快でない　）

　⑩ **て**　　（　不快　・　不快でない　）

　⑪ **あし（脚＋足）**　（　不快　・　不快でない　）

**【異性】**

① **あたま**　（　不快　・　不快でない　）

　② **かお**　　（　不快　・　不快でない　）

　③ **くび＋かた**（　不快　・　不快でない　）

　④ **むね**　　（　不快　・　不快でない　）

　⑤ **おなか**　（　不快　・　不快でない　）

　⑥ **せなか**　（　不快　・　不快でない　）

　⑦ **こし**　　（　不快　・　不快でない　）

　⑧ **おしり**　（　不快　・　不快でない　）

　⑨ **うで（上腕＋前腕）**　（　不快　・　不快でない　）

　⑩ **て**　　（　不快　・　不快でない　）

　⑪ **あし（脚＋足）**　（　不快　・　不快でない　）

質問は以上になります。ご協力ありがとうございました。
